# Sensory neuropathy in young people with type 1 diabetes: a systematic review

**DOI:** 10.1186/1687-9856-2015-S1-O34

**Published:** 2015-04-28

**Authors:** Michelle Jayasuriya, Kim Donaghue, Maria Craig

**Affiliations:** 1 School of Women’s and Children’s Health, UNSW Medicine, University of New South Wales, Sydney, NSW, Australia; 2 Institute of Endocrinology and Diabetes, The Children’s Hospital at Westmead, Sydney, NSW, Australia; 3 Discipline of Paediatrics and Child Health, University of Sydney, Sydney, NSW Australia

## 

Peripheral sensory neuropathy and its risk factors are well-described in adults with type 1 diabetes (T1D). While clinically evident neuropathy is rare in young people with T1D, we and others have shown that subclinical peripheral neuropathy is common [1]. However, the prevalence of sensory neuropathies may be underestimated, due to a lack of established testing guidelines.

We performed a systematic review of the epidemiology of peripheral sensory neuropathy, and diagnostic accuracy of tools used for its assessment, in young people with T1D. We searched Medline and Embase from Jan 1985 to Mar 2014. Inclusion criteria were studies in young people with diabetes duration > 1 year which tested for sensory neuropathy using nerve conduction velocity (NCV), temperature perception threshold (TPT), vibration perception threshold (VPT) and/or clinical examination.

We identified 26 eligible studies, involving 5527 young people with T1D. Only 7/26 (27%) studies were of good methodological quality (Newcastle Ottowa Scale score > 7). Clinical examination yielded a wide variation in the rates of sensory neuropathy, with pooled prevalence of 15% (95% CI 13 to 17). Abnormal VPT was more common, with pooled prevalence 33% (30 to 36), as was TPT, with pooled prevalence 32% (26 to 39). The prevalence of abnormal NCV was similar 36% (33 to 39). Overall, the pooled prevalence of sensory neuropathy, using any test, was 26% (24 to 27). We calculated sensitivity and specificity of the different diagnostic tests that were used in the included studies, in comparison with neuropathies detected by ‘gold standard’ nerve NCV testing. The sensitivity of VPT ranged from 29-62%, and specificity from 65-100%. Sensitivity of TPT was 19% and specificity was 65% (one study). The sensitivity of clinical examination ranged from 0-100% and specificity from 81-100%.

In conclusion, there is marked variation in reported prevalence rates of sensory neuropathy, due to lack of consensus on its definition, classification of abnormal results, and testing methodology. Clinical examination demonstrates the lowest sensitivity for detection of sensory neuropathy in young people with T1D.
